# Environmental sustainability through green HRM: Measuring the perception of university managers

**DOI:** 10.3389/fpsyg.2022.1007710

**Published:** 2022-11-14

**Authors:** Ishfaque Ahmed Lashari, Qiyuan Li, Qamaruddin Maitlo, Faraz Ali Bughio, Ashique Ali Jhatial, Obed Rashidi Syed

**Affiliations:** ^1^School of Management, Wuhan University of Technology, Wuhan, China; ^2^School of Management, China West Normal University, Sichuan, China; ^3^Department of Business Administration, Sukkur IBA University, Sukkur, Pakistan; ^4^Botswana International University of Science and Technology, Palapye, Botswana; ^5^Institute of Commerce, University of Sindh, Jamshoro, Pakistan

**Keywords:** GHRM, employee innovative work behavior, environmental sustainability, RBV, private universities, innovation

## Abstract

Environmental sustainability has gained great momentum worldwide especially in the United Nations (UN), governments, and corporations, and by those who promote global awareness of environmental challenges and are engaged in environmental management. Even as these stakeholders struggle hard, academia has actively engaged in an ongoing debate to make “green human resource management” an independent field of research and teaching. From the large body of academic literature, it has been observed that the field is yet in its embryonic stage in many developing countries such as Pakistan and there is insufficient knowledge on how universities face and manage environmental challenges. Hence, this study addressed this gap in the literature and measured the perception of public and private sector university managers regarding environmental sustainability by using a multi-respondent multi-wave design and collected data from academic heads/supervisors and university faculty in three-time intervals. The data found support for all the hypothetical relationships. The study revealed that the green human resource management (GHRM) practices of public and private universities have a positive impact on environmental sustainability through the mediation of innovative work behavior of employees.

## Introduction

Environmental sustainability has become one of the most critical factors for the existential survival of the planet and it has gained great momentum worldwide, especially in the UN member states. Governments, environment campaigners, corporations, consumers, and non-government organizations have paved the way for global awareness of environmental challenges and their management (Gençay et al., 2018; Kanstrup et al., 2018; Quesada et al., 2018). The 10 principles of the United Nations Global Compact (UNGC; seven to nine derived from the Earth Summit 1992) lay down guidelines for individuals, governments, and especially business corporations to promote environmental sustainability by incorporating strategies, policies, procedures, and establishing a culture of integrity to respond to challenges faced by people and the planet. The United Nations Environment Program (UNEP) recently published a report highlighting that since 1972 it has entered into 1,100 agreements with governments to institute environmental laws and administrative frameworks to address environmental challenges. Nevertheless, the report suggested that despite the huge number of agreements there has been dismal progress with very weak institutional arrangements, lack of coordination, corruption, and poor enforcement by the stakeholders (UNEP, 2019). The findings of the UNEP (2019) report pose new challenges to governments, the corporate sector, academia, and research scholarship to examine the role of governments, businesses, and universities.

In this connection, many researchers think that organizations need to redefine variables of organizational culture and strategy such as mission, vision, and values to inculcate employees' beliefs, attitudes, behaviors, and decision-making to focus on environmental sustainability through green human resource management (Masri and Jaaron, 2017; Menezes et al., 2017; Raut et al., 2017; Zaid et al., 2018). More specifically, Jabbour (2013, p. 147) defined green human resource management (GHRM) as “the systematic and planned alignment of typical HRM practices (such as job analysis, recruitment and selection, training, performance appraisal, and rewards) with the organization's environmental goals.”

GHRM plays a central role in environmental management since human resource policies and practices can integrate with an organization's environment-friendly corporate goals (Renwick et al., 2015; Gholami et al., 2016; Sehnem, 2019; Anwar et al., 2020). Thus, far, GHRM has largely been studied in the context of business corporations and there is very limited evidence of studies on how public and private higher education institutions (HEIs) perform on GHRM-related issues across the world, particularly in developing countries. More recently, Anwar et al. (2020) found the significant impact of GHRM on organizational citizenship behavior (OCB) and environmental performance in two university campuses in Malaysia. However, no substantial evidence is available on whether employees in HEIs are encouraged to use their innovative work behavior (IWB) to mitigate environmental challenges.

Literature indicates that individual behavior plays a significant role in the implementation of environmental policies for sustainability (Lülfs and Hahn, 2013; Tosti-Kharas et al., 2017). Employees' innovative work behavior (IWB) is considered an antecedent of organizational success (Van de Ven, 1986; Woodman et al., 1993; Janssen et al., 2004; Cohen et al., 2012) and it is an intentional effort to introduce and apply new ideas, work methods, and products (Yuan and Woodman, 2010). Therefore, the role of IWB as an intervening variable is very important and hence needs to be studied thoroughly.

Against this backdrop, this study contributes empirical evidence on how university managers i.e., heads of academic and administrative departments perceive the impact of GHRM on environmental sustainability (ENS). In doing so, the present study aims to address the following research gaps:

1. UNGC principles suggest that business corporations consume natural resources, make a profit, and possess abundant resources including human resources. Therefore, drawing on the resource-based view (RBV) theory, we assert that organizations can make a difference by generating environment-specific competencies through the use of unique resources and capabilities, such as GHRM and IWB, to achieve environmental sustainability (Wernerfelt, 1984). This assertion of ours also finds support from the past literature (Hitt et al., 2011; Xie et al., 2019).
2. The present study extends the previous work of Yong et al. (2020) by examining GHRM and environmental sustainability (ENS) relationship and using IWB as an intervening variable. In doing so, we respond to Yong et al. (2020) in several ways: first, we extend the existing model by incorporating IWB as a mediating variable. Second, following their recommendations we collected empirical evidence from the service sector, universities in particular. Third, we used a time lag design to examine the causal relationship among modeled variables. Fourth, Yong et al. (2020) suggested including other variables such as environmental knowledge and awareness, top management commitment, pro-environmental behavior, relative advantage, and green intellectual capital as mediators. We, additionally propose IWB as a potential mediating variable because its nature is very similar to the other suggested variables and has a potential mediating impact on the relationship between GHRM and ENS.
3. Previous literature indicates that individual behavior plays a significant role in the implementation of environmental policies for sustainability (Lülfs and Hahn, 2013; Tosti-Kharas et al., 2017). Thus, we argue that GHRM practices alone will not be sufficient to have an unparalleled influence on an organization's environmental sustainability. Therefore, it has to be complemented with individual behavior that could extend support in this regard. We suggest IWB because it is considered an antecedent of organizational success (Van de Ven, 1986; Woodman et al., 1993; Janssen et al., 2004; Cohen et al., 2012), and it embodies intentional efforts to introduce and apply new ideas, work methods, and products (Yuan and Woodman, 2010). Thus, we infer that IWB will fill this gap by playing an intervening role in the relationship between GHRM and ENS.
4. Jeronimo et al. argued that GHRM practices help organizations gain sustainability through employee engagement. However, what they failed to explain are the means to engage employees through GHRM. We assume that one of the best ways is for organizations to promote sustainability and GHRM through IWB and this may further help organizations to create and maintain a green culture (Jabbour, 2011). Previous studies by Chew and Sharma (2005) and Gerhart and Fang (2005) also maintained that HRM plays a central role in the development of effective organizational culture.
5. The current study responds to the call by Jeronimo et al. to investigate the influence of GHRM on sustainability in traditional organizations that do not prioritize sustainability. Therefore, we expect that our sample from universities (that are traditional in nature with regards to environmental sustainability) will add value to the ongoing debate on environmental sustainability, particularly in educational settings, given that sustainability has been seen in a highly “anthropocentric” and “compartmentalized” manner (Lozano and Huisingh, 2011).
6. Despite empirical evidence on GHRM from Asian countries including Pakistan (Kim et al., 2018; Ahmed et al., 2021; Pham et al., 2019; Anwar et al., 2020), we rarely find studies on GHRM and ENS. Thus, the present study will be among the earliest in this regard. This is important as Renwick et al. (2013) also endorse that GHRM practices will help in achieving sustainability-related goals. More specifically, in proposing the mediating role of IWB between GHRM and ENS, the present study responds to a recent call by Paillé et al. (2020) for such an investigation in the service sector after incorporating mediating variable(s).

## Hypothesis development and conceptualization

Green human resource management, as a construct, has received considerable attention in the recent past (Tang et al., 2017; Kim et al., 2018; Ren et al., 2018; Zaid et al., 2018; Wikhamn, 2019). It has been defined as HRM functions that are planned and aligned with the organization's environmental strategy (Jabbour, 2013). However, scholars have put forward different opinions on the conceptualization of GHRM (Renwick et al., 2013; Opatha and Arulrajah, 2014; Masri and Jaaron, 2017; Nejati et al., 2017; Ren et al., 2018; Kim et al., 2018; Zaid et al., 2018; Wikhamn, 2019). For example, Renwick et al. (2013) defined GHRM as an aspect of human resource management. Whereas, Opatha and Arulrajah (2014) termed GHRM as a means to an end whereby employees are turned into green employees for the accomplishment of environment-related goals. Along similar lines, Masri and Jaaron (2017) suggested that GHRM is a method of reinforcement for employees. Putting it together, GHRM makes employees conscious with respect for the environment and it empowers them (Jabbour, 2013; Nejati et al., 2017; Kim et al., 2018) and through that organizations can achieve their objectives related to the environment. Acknowledging the prominent role of GHRM, the present study used green job analysis, green recruitment and selection (R and S), green training, green performance assessment, and green rewards to measure GHRM (Jabbour, 2011; Yong and Mohd-Yusoff, 2016; Pham et al., 2019). The literature suggests that these factors are essential because they play a significant role in enhancing environmental performance and ultimately leading to environmental sustainability (Pham et al., 2019). For example, a green job analysis helps in determining how employees can be involved in environmental activities, and it also helps to assess knowledge with regard to environmental management (Jabbour et al., 2010; Jabbour, 2011; Yong and Mohd-Yusoff, 2016; Shah, 2019). Similarly, according to Renwick et al. (2013), organizations that care about the environment, are able to brand themselves better, establish a great reputation and play significant roles in recruitment drives. Jabbour et al. (2010, p. 1,057) suggested that green selection is the process of selecting key talented people who show profound commitment to the environment. Training is considered one of the organizational investments for human resource development (Jackson et al., 2011). Organizations have begun training their employees with the required knowledge about environmental policies and practices (Madsen and Ulhoi, 2001; Jabbour et al., 2010, p. 1,057). Green training is now widespread and educates employees on environmentally-friendly business practices (Phillips, 2007). Similarly, Jabbour et al. (2010, p. 1,057) noted that green performance management evaluates employees' environmental performance and the organization's feedback to prevent undesirable attitudes. Likewise, Jackson et al. (2011) found that organizational feedback on employees' environmental tasks could continuously improve environmental management. Furthermore, there is an increasing consensus in the academic literature on green rewards as a strong motivator. Financial and non-financial rewards equally incentivize and help retain key talented manpower at work (Jabbour et al., 2010, p. 1,058; Jackson et al., 2011). We, therefore, aim to investigate the relationship between GHRM and environmental sustainability. The reasons for this hypothesis are as under.

First, Pham et al. (2019) recommended a comparison of GHRM applications and their role in different national contexts. Especially developing countries, because the evidence from these countries is very limited (Baughn et al., 2007; Yong et al., 2020). Second, past literature posits that GHRM plays a pivotal role in various individual-level factors such as affecting employee attitudes and behaviors as well as organizational-level factors such as firm performance in relation to the environment (Ajzen, 1991; Jabbour et al., 2010; Katou and Budhwar, 2010; Daily et al., 2012). Thus, we argue that GHRM enables ENS; our assertion for such support is also in line with Ren et al. (2018) who suggested that GHRM practices will enhance various positive work outcomes at the individual level and the firm level (such as enhanced organizational reputation). More specifically, previous studies that examined the relationship of GHRM with the overall sustainability of the organization have overlooked the specific context of environmental sustainability (Shafaei et al., 2019; Yong et al., 2019). Similarly, Adjei-Bamfo et al. (2019), Hameed et al. (2019), Kim et al. (2018), Pham et al. (2019), and Anwar et al. (2020) looked at the impact of GHRM on environmental performance. Third, according to Jackson and Seo (2010) organizations achieve environmental sustainability by utilizing HRM practices. Thus, HRM becomes an enabler of environmental sustainability. More, recently, Yong et al. (2020) examined a direct link between GHRM and sustainability in manufacturing firms in Malaysia and found empirical support for this relationship. However, there is insufficient evidence available to confirm the relationship of green HRM with environmental sustainability, especially in higher education institutions which are yet to be explored empirically. Specifically, we aim to follow up on the recommendation of Pham et al. (2019) to produce evidence on GHRM and environmental sustainability relationships in the service sector with a specific focus on universities. This is important because past literature has primarily focused on and produced overwhelming evidence from the manufacturing sector alone (Ren et al., 2018; Pham et al., 2019). Lastly, the available evidence on the direct link between GHRM and environmental sustainability limits its use in cross-sectional designs. Therefore, using a time lag design the present study aims at producing more robust empirical evidence on the relationship between GHRM and ENS. Therefore, we propose,

*H*_1_*: GHRM is positively related to environmental sustainability*.

Many authors recognize the importance of employees' innovative work behavior and consider it an antecedent of organizational success (Van de Ven, 1986; Woodman et al., 1993; Janssen et al., 2004). Yuan and Woodman (2010) defined “innovative behavior as an employee's intentional introduction or application of new ideas, products, processes, and procedures to his or her work role, work unit, or organization.” The authors also define innovative work behavior as employees' eagerness to know more, search out technology, advise new ways and means, and investigate and secure resources to implement new ideas.

Recent studies have indicated that various factors such as organizational climate (Carlucci et al., 2020), openness to experience, extraversion and conscientiousness personality (Zuraik et al., 2020), transformational leadership, trust, work engagement (Li et al., 2019), inclusive leadership (Javed et al., 2017), organizational procedural justice (Kim and Park, 2017), and management support (Shalley et al., 2004; Parker et al., 2006; Song et al., 2012) affect innovative work behavior. Fawehinmi et al. (2020) examined the positive relationship between GHRM and employee green behavior with a sample from Malaysian public research universities. Their findings indicate that GHRM significantly affects employees' green behaviors. However, the findings of that study were based on a cross-sectional design thus allowing future researchers to further examine this relationship based on a longitudinal method.

Innovative work behavior implies future-oriented behaviors (Parker et al., 2006). Thus, we assume that employees learn this orientation due to GHRM practices. One reason for this association is the continuous nature of IWB which obtains support from GHRM practices (Parker et al., 2006). Secondly, the literature also explains that the perception of a supportive and encouraging organizational environment becomes the basis of enhanced IWB (Amabile et al., 1996; Zhou and George, 2001). Thus, we argue that GHRM enables a sense of continuous support and encouragement among employees that ultimately becomes the basis for the enhancement of IWB. Moreover, past literature also depicts that human resource practices enhance ability, motivation, and opportunity (Lepak et al., 2006; Sun et al., 2007) for employees. Thus, we assume that GHRM will enhance IWB. This is true because the socio-political perspective of a job indicates that an employee's IWB is a result of the expectations of other stakeholders (managers and coworkers; Daft, 1978; Ashford et al., 1998).

More importantly, behavior is a result of various reinforcements; we assume that GHRM plays the role of reinforcement because it sets a work context that demands employees to be innovative and it provides environment-based support for innovation that manifest IWB (Amabile, 1988; Kanter, 1988; Scott and Bruce, 1994). Our assertion that GHRM will enable employees to demonstrate IWB is in line with Farr and Ford's (1990) argument that if the norms of an organization favor innovation, employees are likely to follow.

According to Jabbour (2015), GHRM operates at three levels (reactive, preventive, and proactive). We assume that IWB is a result of proactive behavior because as highlighted by Jabbour (2015), it is the result of human effort. Lastly, we propose the relationship between GHRM and IWB because Pham et al. (2019) suggested that the application of GHRM should be examined alongside work attitudes and behaviors. Thus, we propose the following hypothesis:

*H*_2_*: GHRM is positively related to innovative work behavior*.

Previous research identified that innovative work behavior helps organizations to succeed in a competitive environment (Kanter, 1983; West and Farr, 1989). An increasing body of knowledge shows IWB has been studied with factors such as organizational culture and climate (Scott and Bruce, 1994), relationship with supervisors (Janssen and Van Yperen, 2004), job characteristics (Oldham and Cummings, 1996), group context (Munton and West, 1995), and individual differences (Bunce and West, 1995). These studies have identified that IWB can be determined by an employee's knowledge, skills, and capability.

There is enough evidence with regard to what causes innovative work behavior (Oldham and Cummings, 1996; Janssen and Van Yperen, 2004; Shalley et al., 2004; Parker et al., 2006; Song et al., 2012; Javed et al., 2017; Kim and Park, 2017; Li et al., 2019; Carlucci et al., 2020). Moreover, scholars have also identified IWB as an antecedent of organizational success (Woodman et al., 1993; Janssen et al., 2004). Notwithstanding these studies, scholars have ignored the important role of IWB in influencing other work outcomes. Particularly, we assume that IWB plays a significant role in environmental sustainability. This is true because IWB is an intentional attempt by employees to produce new ideas, introduce work methods that are efficient, and propose new processes and products (Yuan and Woodman, 2010). Thus, drawing on the definition of IWB by Yuan and Woodman (2010), we argue that IWB will lead organizations toward environmental sustainability.

Past scholars have examined three pillars of sustainability, namely, economic, social, and environmental (DuBois and Dubois, 2012). However, scholars suggest that among these ENS provides long-term benefits to organizations as a whole (DuBois and Dubois, 2012). Friedman (1970) suggested that ENS can be obtained by utilizing organizational resources efficiently and effectively. Thus, we assume that IWB, by producing innovative processes, work methods, and products, paves a new way for ENS. Secondly, we also assume that environmental sustainability is a result of proactive behavior (Kolk, 2008; Ones and Dilchert, 2010; Wensen et al., 2011). Thus, we argue that IWB will produce ENS.

Our third justification for the hypothetical relationship between IWB and ENS is based on previous work. For example, Omri (2015) suggested that IWB and firm performance are significantly related to each other, and Iqbal et al. (2018) found that employees' green behavior is positively related to ENS. However, previous studies have overlooked innovative work behavior of employees could be eco-friendly and may associate with environmental sustainability. As a result, this study tends to hypothesize as below:

*H*_3_*: Employees' innovative work behavior is positively related to environmental sustainability*.

Sustainability has gathered great momentum and corporate managers have had to invest huge portions of their company's profits in environmental sustainability (Chouinard et al., 2011; Yong et al., 2019). The triple bottom line principle (people, planet, and profit) is the true spirit of economic, environmental, and social sustainability (Elkington, 1997). Environmental sustainability is the impact of business on the environment. Most governments the world over have instituted legal-administrative frameworks which require public, private, and multinational corporations to comply with the set standards and protocols of environmental management (Jabbour and Santos, 2008). In the same vein, Wirtenberg et al. (2007) observed that the HRM department, already considered a change agent and development partner in every organization, could also usher organizations to develop environment-friendly competencies, collaborative strategies, and organizational capabilities to achieve environmental sustainability. Taylor et al. (2012) advise HRM could lead at the organizational level by developing environmentally sustainable protocols embodied in policy and practice to pave the way for the socioeconomic wellbeing of the firm, its people, and society. Employees innovate their behavior by developing their ideas or borrowing from others' good practices to accomplish organizational eco-policies and create a green corporate culture. Jabbour and Santos (2008) observed that HRM can encourage employees to use innovative behavior to inspire great environmental performance. Thus, we assert from past studies that GHRM affects ENS through innovative work behavior because HRM affects organizational effectiveness indirectly (Collins and Smith, 2006; Kase et al., 2009).

Recently, Yusliza et al. (2015) observed that organizations are aligning their HRM with three sustainability pillars economic, environmental, and social balance. More recently, a few researchers have explored GHRM and environmental performance (Kim et al., 2018; Hameed et al., 2019; Anwar et al., 2020). Others have studied GHRM and employee green behavior (Fawehinmi et al., 2020), and various other factors that support and enhance IWB (Amabile et al., 1996; Zhou and George, 2001). Based on these findings from the literature we assert that, first, there is a paucity of research on the relationship between GHRM and ENS; though recently Yong et al. (2019) examined the linkage of GHRM with sustainability in the Malaysian context. However, GHRM and ENS remain unexplored.

Second, despite widespread attention to achieving environmental sustainability through GHRM, the mediating effect of IWB has yet not been studied (Aguinis and Glavas, 2012). We assert that GHRM, ENS, and IWB are all proactive in nature and are a result of human effort (Jabbour, 2015). Thus, we assume that IWB will mediate the relationship between GHRM and ENS. In addition, past literature posits that GHRM plays a pivotal role in various work factors such as affecting employee attitudes and behaviors as well as organizational level factors such as firm performance in relation to the environment (Ajzen, 1991; Chen, 2008; Jabbour et al., 2010; Katou and Budhwar, 2010; Daily et al., 2012). Thus, we argue that GHRM will enhance IWB which will ultimately lead toward ENS. Third, in proposing the mediating role of IWB between GHRM and ENS, the present study responds to a recent call by Paillé et al. (2020) who have suggested such an investigation by incorporating mediating variable (s) in the service sector. Fourth, we aim to bring empirical evidence on the relationship between GHRM and ENS through the mediating effects of IWB from developing countries (Baughn et al., 2007; Pham et al., 2019; Yong et al., 2020). Fourth, as recommended by Pham et al. (2019), the current study specifically will explore the said relationship in the service sector as past research was largely focused on the manufacturing sectors. Lastly, using a time lag design, the current study addresses the limitation of the past studies that have examined the GHRM and ENS relationship. As a result, the study hypothesizes the following:

*H*_4_*: Innovative work behavior of university employees significantly mediates the relationship between GHRM and environmental sustainability*.

## Theoretical underpinning

**Figure 1:**
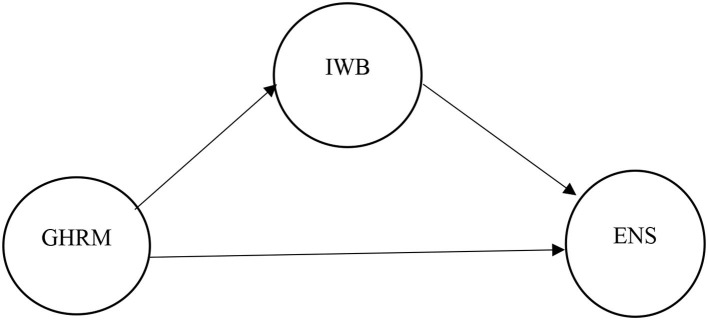
Conceptual framework.

The literature review based on previous studies indicates the use of various theories while explaining the critical role of GHRM. Among these, the most popularly used theories are the ability-motivation-opportunity (AMO) theory, the theory of planned behavior, the social exchange theory, the social identity theory, and the resource-based view theory (RBV; Pham et al., 2019; Amrutha and Geetha, 2020). The final selection of a theory was based on the operationalized concept of GHRM and study objectives. Among these theories, RBV makes the widest use due to its open nature and relevance to GRHM. Thus, drawing on the resource-based view theory (RBV), the present study proposes the mediating role of innovative work behavior (IWB) in the relationship between GHRM and environmental sustainability as shown in Figure 1.

We present the following theoretical justifications for the use of RBV (Wernerfelt, 1984; Barney, 1991). First, as suggested by Wernerfelt (1984), organizations generate competencies through the use of unique resources and capabilities and this leads them toward the achievement of sustained competitive advantage. Thus, we assume GHRM and IWB as a resource and capabilities that help organizations achieve sustained competitive advantage which in this case is environmental sustainability. Our assertion of the important role of GHRM and IWB as resources and capabilities finds support in the work of Xie et al. (2019) who stated that resources and capabilities are critical for gaining a competitive advantage. Moreover, according to Hitt et al. (2011), a firm's success largely relies upon its capabilities and resources. This is important because organizations have to comply with external and internal pressures thus generating resources and capabilities and making the right use of these resources becomes essential for organizations. Therefore, we argue that organizations that make the right use of GHRM and promote IWB ultimately achieve environmental sustainability. Secondly, one of the prominent assertions of RBV is that organizations invest in practices and processes that could help them stand unique among their competitors (Dey et al., 2019; Schedlitzki, 2019). We assume GHRM as a resource enables organizations to generate such practices, like IWB, that lead them to gain differentiation and a competitive advantage such as ENS. This is significant because RBV further explains that particular internal resources of an organization are of important value because these contribute toward the success of an organization and its sustainability (Wright and Geroy, 2001). Therefore, our argument for using GHRM as a resource that enables ENS through IWB finds support from previous studies (Huselid, 1995; Koch and McGrath, 1996; Gholami et al., 2016; Yong et al., 2019). Particularly, Jabbour and Santos (2008) examined HR practices and sustainability through RBV. Thus, the present study seeks support from RBV for this framework.

Lastly, previous research has established environmental regulations, green culture, and green innovation strategies as resources that help organizations gain a competitive advantage that competitors find difficult to imitate (Chiou et al., 2011). Thus, we assume that GHRM and IWB, when reinforced appropriately, give organizations similar strength. Lastly, our claim for using GHRM as a means to transform manpower into valuable and inimitable resources that can be a competitive advantage for firms is in line with various studies (Barney, 1991; De Saá-Pérez and García-Falcón, 2002; Barney et al., 2011; Ahmad, 2015; Longoni et al., 2016; Masri and Jaaron, 2017; Yusoff et al., 2018; Zaid et al., 2018). Therefore, we propose IWB as a mediating mechanism to explain the relationship between GHRM and ENS.

## Research methods

### Sample and procedure

The present study aims to determine the relationship between GHRM and environmental sustainability and whether university managers utilize innovative work behavior to mitigate environmental challenges. Therefore, university managers were selected as respondents for the study. Public and private universities from Pakistan's Sindh province were of special interest to the researchers as they are sensitive to environmental issues, use formal HRM functioning, and are also subject to government environmental laws. We targeted respondents at all management levels who had structural authority, responsibility, and knowledge of the sensitivity of the environmental issues concerned.

A multi-respondent multi-wave design was used for data collection to separate cause and effect for a period of 4 months. We collected data for all the latent variables at three different time points. First, data regarding green HR practices were collected from university faculty (T1). After a 4-month interval (T2), we approached the heads of departments/academic heads who supervise faculty directly, to collect data regarding the IWB of faculty members. Lastly, we collected data about ENS (T3) from university faculty.

The reason to do the study at three different times was to establish a causal relationship between data points since it required matching supervisor-subordinate data collected *via* surveys, therefore, the time-lag method was used to robust the data quality.

Before starting the data collection process, we obtained approval from the ethical committee of the lead author's university. About 500 questionnaires were distributed to the heads of academic and administrative departments (HoDs) who actively participated in HRM functioning in their respective universities and they were also requested to connect with faculty members. At the start of data collection (T1), we explained the nature and objectives of the study and highlighted the strict confidentiality of respondent information. We also sought consent from the participants to participate in the T-2 and T-3 waves of our survey and only those respondents who agreed to participate in the future were contacted at a later stage. We received a total of 260 supervisor-subordinate matched responses out of which 223 were usable.

There were 183 (82.1%) male respondents and 40 (17.9%) female respondents. Of the respondents, 188 (84.3%) were married and 35 (15.7%) were single. The majority (122, 54.7%) were aged between 41 and 50, and 121 (54.3%) had Ph.D. qualifications. Most of them (154, 69.1%) had between 11 and 20 years of experience. The majority (118, 52.9%) of respondents held middle-level management positions while 102 (45.7%) were in first-line management. About 70.8% (158) were from public universities and the balance 29.1% (65) were from private universities.

## Measurements

Green HRM practices were measured against five dimensions: green job analysis with three items, green R and S with four items, green training with three items, green performance assessment with three items, and green rewards with two items. These measurement items were adapted from Jabbour (2011) and Yong and Mohd-Yusoff (2016). Innovative work behavior was measured using five items. The scale was borrowed from Scott and Bruce (1994) who reported a Cronbach alpha of 0.93. The innovative behavior scale has been used several times and it measures innovative behavior adequately (Yuan and Woodman, 2010). Environmental sustainability was measured using a five-item scale adapted from Zhu et al. (2008), Laosirihongthong et al. (2013), and Paulraj (2011). A seven-point Likert-type scale ranging from 1 (not at all) to 7 (to a very great extent) was applied in answer to each item.

## Results and discussion

### PLS-SEM analysis

To analyze the research model developed for this study, the structural equation modeling (SEM) technique i.e., partial least squares (PLS) package SmartPLS 3.2.8 was utilized (Ringle et al., 2015). SmartPLS is a second-generation statistical package that is compatible to perform adequate analysis on smaller sample sizes with non-normal dataset (Qalati et al., 2022a). We applied a two-stage approach; in the first stage, we tested the measurement model, followed by an examination of the structural model at stage two (Anderson and Gerbing, 1988; Hair et al., 2017). In the first stage, we tested indicator and internal consistency reliability and convergent and discriminant validity (Henseler et al., 2009; Hair et al., 2014, 2017). In the second stage, we performed a bootstrapping procedure of PLS-SEM with a resampling rate of 5,000. The main objective of doing bootstrapping was to obtain path coefficients including the beta values, standard error, *t*-values, *p*-values, and bootstrapped confidence intervals (Qalati et al., 2022b).

### Analysis of measurement model

We followed Hair et al. (2010) approach to evaluate the measurement model by performing various tests such as individual item reliability, internal consistency, content validity, convergent validity, and discriminant validity. In the initial stage, the factors loadings of IWB4 were found below the threshold and as a result, this indicator was deleted.

#### Individual item reliability

Duarte and Raposo (2009), Hair et al. (2014); and Hulland (1999) advised the rule of thumb to retain an item whose loading falls between 0.40 and 0.70. Individual item reliability was assessed from the outer loadings of each of the measures (items) of each construct (Hair et al., 2012, 2014). The outer loadings for each of the latent variables of the present study were sufficiently up to 0.60, except IWB4, which was deleted. Therefore, the present study successfully met the individual item reliability criteria (refer to Table 1).

**Table 1:** Measurement model.

**Construct**	**Item**	**Loadings**	**CR**	**AVE**
ENS	ENS1	0.768	0.863	0.559
	ENS2	0.829		
	ENS3	0.669		
	ENS4	0.721		
	ENS5	0.740		
GJA	GJA1	0.744	0.784	0.548
	GJA2	0.689		
	GJA3	0.786		
GP	GP1	0.761	0.839	0.636
	GP2	0.856		
	GP3	0.772		
GRS	GRS1	0.802	0.853	0.594
	GRS2	0.845		
	GRS3	0.662		
	GRS4	0.763		
GRW	GRW1	0.820	0.831	0.711
	GRW2	0.866		
GT	GT1	0.833	0.855	0.665
	GT2	0.888		
	GT3	0.715		
IWB	IWB1	0.853	0.852	0.599
	IWB2	0.864		
	IWB3	0.818		
	IWB5	0.506		

ENS, environmental sustainability; GJA, green job analysis; GP, green performance; GRS, green recruitment and selection; GRW, green rewards; GT, green training; IWB, innovative work behavior.

#### Internal consistency reliability

To assess internal consistency reliability i.e., composite reliability Bagozzi and Yi (1988) and Hair et al. (2011) advised coefficient of CR should be 0.70 or above. Table 1 exhibits CR coefficients for each of the latent variables ranging from 0.784 to 0.863. The values in Table 1 suggest that all the values load pretty well above the cut-off values which confirms the internal consistency reliability of the measures (Bagozzi and Yi, 1988; Hair et al., 2011).

#### Convergent validity

According to Fornell and Larcker (1981) convergent validity can be assessed by looking into the values of average variance extracted (AVE). Similarly, Chin (1998) noted that the AVE scores should be at least 0.50 or larger to confirm the convergent validity of a particular construct. The AVE scores provided in Table 1 indicate that all the constructs of the present study achieved the minimum threshold values of 0.50. As a result, it is concluded that the study demonstrated adequate convergent validity (Chin, 1998).

#### Discriminant validity (DV)

To analyze the discriminant validity, we used the Heterotrait-Monotrait criterion (HTMT). The HTMT ratio of correlations was used to assess the DV which is considered a conservative approach for assessing DV (Henseler et al., 2015). DV refers to the “extent to which a construct is truly distinct from other constructs by empirical standards” (Hair et al., 2017, p. 104). More recently Hair et al. (2019) advised that the threshold value should not be more than 0.90. An HTMT value above 0.90 highlights a lack of DV that shows that the constructs are conceptually identical. Table 2 presents details that indicate that HTMT was established at HTMT 0.90; all constructs have HTMT scores < 0.90. This indicates that all constructs of the present study measure distinct domains. Additionally, the results of bootstrapping indicate HTMT values were significantly lower than 1, thereby confirming the DV (Hair et al., 2017). Table 3 indicates that discriminant validity was established (Kline, 2011; Henseler et al., 2015).

**Table 2:** Discriminant validity Heterotrait-Monotrait ratio (HTMT).

**Constructs**	**1**	**2**	**3**	**4**	**5**	**6**
ENS						
GJA	0.288					
GP	0.454	0.735				
GRS	0.759	0.362	0.443			
GRW	0.497	0.587	0.650	0.504		
GT	0.474	0.441	0.541	0.539	0.770	
IWB	0.594	0.619	0.388	0.597	0.396	0.490

ENS, environmental sustainability; GJA, green job analysis; GP, green performance; GRS, green recruitment and selection; GRW, green rewards; GT, green training; IWB, innovative work behavior.

**Table 3:** Hypotheses testing.

**Hypotheses**	**Relationships**	**Beta**	**SE**	***t*-value**	**P-values**	**Confidence intervals**	
						**5.0%**	**95.0%**
H1	Green-HRM -> ENS	0.421	0.084	5.033	0.000	0.286	0.559
H2	Green-HRM -> IWB	0.515	0.070	7.378	0.000	0.399	0.629
H3	IWB -> ENS	0.274	0.093	2.938	0.002	0.114	0.426
H4	Green-HRM -> IWB -> ENS	0.141	0.048	2.966	0.002	0.061	0.221

### Structural equation model

To test our study model, we performed a bootstrapping procedure in the second stage of PLS-SEM with a resampling rate of 5,000. The main objective of bootstrapping was to obtain path coefficients including the beta values, standard error, *t*-values, *p*-values, and bootstrapped confidence intervals (Hair et al., 2017).

The results of the hypotheses testing presented in Table 4 show that all the hypotheses are supported. We tested the relationship of green human resource management (GHRM) with environmental sustainability (ENS). The results in Table 4 show β = 0.421, *t* = 5.033, *p* < 0.000. Similarly, H_2_ intended to measure the relationship of GHRM with the innovative work behavior (IWB) of university managers. The results in Table 4 show β = 0.515, *t* = 7.378, *p* < 0.000. Likewise, H_3_ hypothesized to measure the relationship between IWB with ENS. The results in Table 4 show β = 0.274, *t* = 2.938, *p* < 0.002. The last hypothesis aimed to measure the mediating relationship among GHRM -> IWB-> ENS. The results show that mediation exists (β = 0.141, *t* = 2.966, *p* < 0.002). The *R*^2^ evaluates the predictive power of the model (Hair et al., 2017). According to Cohen (1988), *R*^2^ values of 0.26, 0.13, and 0.02 are deemed as substantial, moderate, and weak, respectively. The values of this study suggest that GHRM and IWB predicted about 37% variance (*R*^2^ = 0.371) in managing environmental sustainability. Additionally, the *R*^2^ value for IWB was 0.265 suggesting that the green HRM practices explained IWB up to 26%.

**Table 4:** Construct cross-validated redundancy.

**Constructs**	**SSO**	**SSE**	***Q* ^2^**
ENS	1,115.000	898.771	0.194
IWB	892.000	758.744	0.149

### Predictive relevance of the model

To evaluate the reflective nature of the endogenous latent variable, we used a cross-validated redundancy measure (*Q*^2^). Many researchers suggest that assessing the predictive relevance of the model is of high importance (Chin, 2010; Ringle et al., 2012; Hair et al., 2013). The predictive relevance is an additional assessment that is recommended because the goodness-of-fit (GoF) index is not suitable for model validation as it cannot separate the valid and invalid models (Henseler and Sarstedt, 2013; Hair et al., 2014). Henseler et al. (2009) stated that in a researcher model where the *Q*^2^-value(s) is found greater than zero, it is considered that the model has a predictive relevance. Table 4 provides the cross-validated redundancy *Q*^2^ test results for ENS and IWB. The cross-validated redundancy value (*Q*^2^) as suggested by Chin (1998), and Henseler et al. (2009) is greater than zero. This suggests that our model successfully demonstrated predictive relevance.

## Discussion

The main objective of this study was to examine whether the functioning of HRM in public and private universities in Sindh is environmentally friendly. The study also aimed to assess whether university managers were encouraging their staff to use innovative work behavior to manage environmental issues in their respective universities. For this purpose, we examined the link between GHRM and ENS, GHRM and IWB, IWB and ENS, and the mediating effect of IWB on GHRM and ENS. These hypothesized relationships found empirical support. The findings of this study are in line with previous studies that examined the role of GHRM practices in environmental sustainability relationships and the role of IWB (Kanter, 1983; West and Farr, 1989; Pfeffer, 2010; Yuan and Woodman, 2010; Paulraj, 2011; Guerci et al., 2016; Masri and Jaaron, 2017; Zaid et al., 2018).

### Theoretical implications

Based on the RBV theory, we proposed that green HRM functions consisting of green job analysis, green recruitment and selection, green training, green performance assessment, and green rewards are strongly linked with environmental sustainability. Also, GHRM paved the way for employees to innovate their work behavior to manage environmental issues. The findings of this study as hypothesized showed a statistically significant and positive relationship between GHRM and environmental sustainability. RBV theory suggests that organizations could have a competitive advantage in human resource and their skills, knowledge, and abilities that cannot be copied. The findings of this study are in line with many scholars who consider RBV theory coupled with HRM practices could transform manpower into valuable and inimitable resources that can be a competitive advantage for firms (Barney, 1991; De Saá-Pérez and García-Falcón, 2002; Barney et al., 2011; Ahmad, 2015; Longoni et al., 2016; Masri and Jaaron, 2017; Yusoff et al., 2018; Zaid et al., 2018).

In light of RBV (Wernerfelt, 1084) we assert GHRM and IWB as resources and capabilities to achieve organizational environmental sustainability. Furthermore, the results of the present study support the notion that resources and capabilities are critical for gaining competitive advantage and a firm's success largely relies upon them (Hitt et al., 2011; Xie et al., 2019). Secondly, the current study employed GHRM as a resource enabler drawing upon the assertion of RBV that organizations invest in practices and processes to make them stand unique (Dey et al., 2019; Schedlitzki, 2019). Our assumption that GHRM will help organizations generate practices and processes that could lead toward differentiation and competitive advantage through IWB found empirical support. We, therefore, believe that GHRM is an internal resource (Wright et al., 1994) that contributes toward environmental sustainability through IWB (Huselid, 1995; Koch and McGrath, 1996; Jabbour and Santos, 2008; Gholami et al., 2016; Yong et al., 2019). Lastly, we argued based on RBV that GHRM and IWB, when utilized appropriately, give organizations a competitive advantage (Chiou et al., 2011) and these factors help organizations in transforming their manpower into valuable and inimitable resources (Barney, 1991; De Saá-Pérez and García-Falcón, 2002; Barney et al., 2011; Ahmad, 2015; Longoni et al., 2016; Masri and Jaaron, 2017; Yusoff et al., 2018).

### Managerial implications

The findings of this study offer far-reaching implications for academics, researchers, managers, and policymakers across the board not only for public and private universities, but also for small and large organizations, for-profit or NGOs, MNCs, and government organizations. A conceptual framework with utmost parsimony presents guidelines across the board to restructure HRM with green practices and allow employees to use innovative work behavior to manage environmental challenges in organizations. This study's findings are also a source of inspiration for educational institutions from primary to tertiary levels to restructure syllabi and train employees with green policies and practices for the larger interest of future managers and society. GHRM practices enhance universities' environmental and social performance as well as may attract more student enrollment and funding from government and environmental organizations.

Government policymakers, especially in Asia, can institute appropriate legal-administrative frameworks that seek organizational compliance and disclosure of environmental sustainability standards and practices. There are umpteen environmental laws and policies of the government of Pakistan, but there is an urgent need to seek organizational compliance and compulsory annual or periodical disclosure not only in Pakistan. This should be made mandatory across Asia, particularly in all developing countries.

In compliance with government laws and policies, organizations need to respond equally by investing in organizational culture that is committed to GHRM practices and encouraging employees to actively participate in environmental sustainability standards by innovating their behaviors. Organizations also need to reward environment-friendly employees with tangible and intangible rewards to persuade other employees and other organizations to follow suit.

The findings of this study offer several implications for management: first, our findings suggest that GHRM works best not only for business organizations but is equally important for the service sector, especially in the context of universities. This implies that universities elsewhere could also restructure their human resource policies and practices in alignment with environmental issues and could respond to managing the environment successfully. Second, based on our results we suggest that the service sector should be cautious about environmental challenges and Asian universities, in particular, should give due importance to environmental sustainability and factors that affect it (such as GHRM, green culture, and IWB). Third, previous studies have established environmental regulations, green culture, and green innovation strategies as resources that help organizations gain a competitive advantage that competitors find difficult to imitate (Chiou et al., 2011). We, therefore, suggest that managers and policymakers need to look into how and when GHRM and IWB could be used to develop green culture and gain such an advantage. Lastly, Jeronimo et al. argued that GHRM practices help organizations gain sustainability through employee engagement. However, what Jeronimo et al. did not explain are the means to engage employees through GHRM and green culture. We assume that the best way for organizations is to promote sustainability and GHRM links through IWB. Thus, managers need to pave ways for encouraging IWB in the workplace.

## Limitations and future research directions

Though the findings of this study appear to be robust and essential, there are certain limitations that stem from the conceptual framework and its implications. Although, all the constructs used in this study are well-grounded in the extant literature which can be considered adequate for studies on GHRM practices in organizations, nevertheless, other factors may also contribute to this framework. As a result, this framework could also be revisited in other business corporate sectors, if possible revised, and additional variables that support GHRM and environmental sustainability could be included for wider generalizability of the model. In this regard, we specially suggest: First, constructs such as green culture, pro-environmental behavior, green commitment, environmental sustainability, and environmental responsibility may be used as intervening variables in the relationship between GHRM and ENS. Second, we recommend qualitative studies on the role of green commitment in enabling the GHRM and ENS relationship. We believe, it is important because in-depth qualitative studies may explore factors that hinder and/or promote the implementation of green initiatives for obtaining ENS. Third, the current study responded to the suggestion by Yong et al. (2020) and collected empirical evidence on GHRM and ENS relationship from the service sector, and universities in particular. However, we believe that such an investigation may be extended to other service industries such as restaurants, hotels, and hospitals for better generalizability of our findings.

## Conclusion

Tertiary education institutions such as universities are fundamental sources of providing competent manpower to industry and employment for families and society. This study attempted to understand to what extent universities in Pakistan are aware of environmental challenges and whether they use green HRM practices and encourage their manpower to use innovative work behavior and adapt to new technology and technique to take care of the environment. After an exhaustive literature review, it was observed that there is an acute shortage of empirical evidence to report how universities across the globe are using GHRM and encouraging employees to use innovative behavior to manage environmental challenges. As a result, this study attempted to fill this gap by undertaking this study in Sindh, Pakistan.

The government of Pakistan has several laws and policies supporting the adoption of environmentally friendly activities. GHRM is one of the significant strategies for organizations to recruit, select, and train staff that is environmental-friendly and is ready to mitigate environmental problems. Several studies have confirmed that GHRM offers organizations several benefits such as reduction of waste and cost, conservation of energy, and attraction and retention of talent (Renwick et al., 2013; Sawang and Kivits, 2014). The corporate sector is already cautious about paying attention to environmental sustainability since natural resources are rapidly depleting. Currently, educational institutions have also begun to manage environmental challenges by applying GHRM and allowing employees to use innovative behavior. Based on the RBV framework, the present study confirms the influence of GHRM practices on environmental sustainability and bridges an important research gap in the literature. Based on the aforementioned discussion, the findings reveal that GHRM along with innovative work behavior of employees leads to environmental sustainability in public and private universities in Sindh, Pakistan. This implies that to achieve environmental sustainability, the HRM department should update traditional practices with green practices and focus on candidates who are cautious of and possess environmental knowledge during the recruitment process. Based on needs-assessments, HRM departments should provide environment-related training such as waste management, recycling, and energy management. Despite the limitations of this study, we hope that this research opens up the debate on GHRM as a field of research and practice and also inspires future research on greening HRM policy and practice for managing environmental challenges across business sectors in the country.

## Data availability statement

The data presented in this article will be made available upon request to the corresponding author/s.

## Ethics statement

Ethical review and approval was not required for the study on human participants in accordance with the local legislation and institutional requirements. Written informed consent from the patients/participants or patients/participants legal guardian/next of kin was not required to participate in this study in accordance with the national legislation and the institutional requirements.

## Author contributions

QL contributed in methodology part. FA have contributed in language correction and review process. All authors listed have made a substantial, direct, and intellectual contribution to the work and approved it for publication.

## Conflict of interest

The authors declare that the research was conducted in the absence of any commercial or financial relationships that could be construed as a potential conflict of interest.

## Publisher's note

All claims expressed in this article are solely those of the authors and do not necessarily represent those of their affiliated organizations, or those of the publisher, the editors and the reviewers. Any product that may be evaluated in this article, or claim that may be made by its manufacturer, is not guaranteed or endorsed by the publisher.
